# Knowledge synthesis and the Canadian Institutes of Health Research

**DOI:** 10.1186/2046-4053-1-6

**Published:** 2012-02-09

**Authors:** Ian D Graham

**Affiliations:** 1 Knowledge Translation and Public Outreach, Canadian Institutes of Health Research, 160 Elgin Street, Ottawa, Ontario, K1A 0W9, Canada

## Abstract

The Canadian Institutes of Health Research (CIHR) is Canada's premier health-research funding agency. We fund nearly 14,000 researchers and trainees in four theme areas: biomedical, clinical, health services, and population and public-health research. Our mandate is 'to excel according to international standards of scientific excellence, in the creation of new knowledge and its translation into improved health for Canadians, more effective health services and products and a strengthened Canadian health care system'. Knowledge synthesis is a key element of the knowledge-translation objectives of CIHR, as outlined in our definition of knowledge-translation.

## Background

The Canadian Institutes of Health Research (CIHR) is Canada's premier health-research funding agency. CIHR funds nearly 14,000 researchers and trainees in four theme areas: biomedical, clinical, health services, and population and public-health research. Our mandate is 'to excel according to international standards of scientific excellence, in the creation of new knowledge and its translation into improved health for Canadians, more effective health services and products and a strengthened Canadian health care system' (http://cihr-irsc.gc.ca/e/7263.html). Knowledge synthesis is a key element of the knowledge-translation objectives of CIHR as detailed in our definition of knowledge-translation.

Knowledge translation is a dynamic and iterative process that includes synthesis, dissemination, exchange, and ethically sound application of knowledge to improve the health of Canadians, provide more effective health services and products, and strengthen the healthcare system. This process takes place within a complex system of interactions between researchers and knowledge users that may vary in intensity, complexity, and level of engagement depending on the nature of the research and the findings, and the needs of the particular knowledge user (http://www.cihr-irsc.gc.ca/e/39033.html).

## Overview

CIHR takes a broad view of the concept of 'synthesis'. It is more than systematic review; it is a family of methodologies that can be deployed to achieve a better understanding of what is known in a given field or what the current gaps in knowledge are. CIHR defines synthesis as '...the contextualization and integration of research findings of individual research studies within the larger body of knowledge on the topic. A synthesis must be reproducible and transparent in its methods, using quantitative and/or qualitative methods. It could take the form of a systematic review, follow the methods developed by the Cochrane Collaboration, result from a consensus conference or expert panel or synthesize qualitative or quantitative results. Realist syntheses, narrative syntheses, meta-analyses, meta-syntheses and practice guidelines are all forms of synthesis' (http://www.cihr-irsc.gc.ca/e/39033.html).

CIHR places considerable value on knowledge synthesis. For example, we require applicants applying for funding to conduct a randomized controlled trial (RCT) to include a systematic review with the proposal to establish the need for the trial, and to contextualize the trial within the already existing body of knowledge. To support the conduct of synthesis, CIHR developed a knowledge synthesis funding program in 2005, which has two intakes a year. To date, this funding program has funded over 175 scoping and full reviews using a variety of methodologies. This programs falls under our category of 'integrated knowledge-translation research' (http://www.cihr-irsc.gc.ca/e/39033.html), which requires researchers to partner with knowledge users (for example, clinicians and policy-makers) on the grant proposal to ensure that the correct synthesis questions are being asked and to work collaboratively to further facilitate the uptake of the results. In 2008, in response to the interest of our provincial and federal ministries of health to have syntheses conducted for them sufficiently quickly to be incorporated into the policy-making process, we developed an expedited knowledge synthesis funding program. Furthermore, the commitment of CIHR to synthesis is evidenced by the provision of national funding to the Canadian Cochrane Centre (http://ccnc.cochrane.org) and the Queen's Joanna Briggs Collaboration (QJBC) for Patient Safety (http://meds.queensu.ca/qjbc). Several of the CIHR institutes have supported the conduct of synthesis by issuing requests for applications or priority announcements to fund syntheses, or engaged the Canadian Cochrane Centre to undertake a synthesis to determine the state of the science in a particular area so as to inform the launch of a request for applications in that area. We have also funded Knowledge Synthesis Canada (http://www.kscanada.ca), a network of researchers focused on synthesis. To support the research community in better understanding the importance of synthesis, CIHR commissioned Dr Jeremy Grimshaw to create an educational module on conducting synthesis and the use of synthesis for informing decision-making (http://www.cihr-irsc.gc.ca/e/41382.html). At CIHR, synthesis is considered an essential tool for supporting knowledge use and decision-making in our health system.

## Discussion

Registration of systematic reviews is of great interest to CIHR, as it provides a mechanism to coordinate and capture the breadth of activities taking place around the globe. As a public funder, having a registry assists us and the research community in avoiding duplication so that our limited research funds can be deployed effectively and efficiently. A registry assists the research community in identifying pre-existing systematic reviews, avoiding duplicate work, and hopefully encouraging work to fill in identified gaps in knowledge. PROSPERO is an example of such a registry, and can be accessed at http://www.crd.york.ac.uk/prospero/.

Furthermore, a registry that captures the current activities and has a minimum dataset of registry elements can help to avoid publication bias and selective reporting. This is achieved by having a permanent record that allows users to compare the methodologies and outcomes with those identified in the protocol. In addition,, in the event that a review is terminated, the reasons for termination and details of the review's unpublished results could be made available.

From a knowledge-translation perspective, not only does registration benefit researchers and funders, but there is immense potential for people who can use the results of syntheses to inform their decision-making. PROSPERO will be an invaluable way of assisting knowledge users to identify the latest version of a synthesis, access up-to-date, scientifically sound, summaries of the evidence, and identify researchers in key topic areas with whom it may be beneficial to establish future collaborations.

## Conclusion

CIHR is currently modifying its funding program criteria for the Knowledge Synthesis Funding Opportunity to require registration of all systematic reviews funded through this mechanism, and we look forward to the day when knowledge syntheses of all types (not just systematic reviews) can be registered.

## List of Abbreviations

CIHR: The Canadian Institutes of Health Research; PROSPERO: International Prospective Register of Systematic Reviews; QJBC: Queen's Joanna Briggs Collaboration; RCT: randomized controlled trial.

## Competing interests

The author declares that they have no competing interests.

## Authors' contributions

Ian D. Graham is the sole author of this manuscript.

